# Transient siRNA-mediated protein knockdown in mouse followed by feeding/starving cycle and liver tissue analysis

**DOI:** 10.1016/j.xpro.2021.100500

**Published:** 2021-04-27

**Authors:** Lidia Wrobel, Farah H. Siddiqi, David C. Rubinsztein

**Affiliations:** 1 Department of Medical Genetics, Cambridge Institute for Medical Research, University of Cambridge, Hills Road, Cambridge, CB2 0XY, UK; 2 UK Dementia Research Institute, Cambridge, UK

**Keywords:** Metabolism, Model Organisms, Molecular Biology, Gene Expression

## Abstract

We present a protocol for in vivo siRNA-mediated knockdown of a gene of interest in mouse liver using systemic delivery via intravenous injection. We describe a step-by-step protocol for delivery of siRNA particles, with tips on how to optimize dosage. We detail steps for feeding/starving cycles as well as for liver tissue isolation, followed by gene expression analysis, measured at the mRNA and protein levels.

For complete information on the generation and use of this protocol, please refer to Wrobel et al. (2020).

## Highlights

• siRNA-mediated knockdown of a gene of interest in mouse liver
• Gene expression analysis at the mRNA and protein levels in the liver tissue
• Fasting/refeeding response at the mRNA and protein levels in the liver tissue

## Graphical abstract

**Figure:**
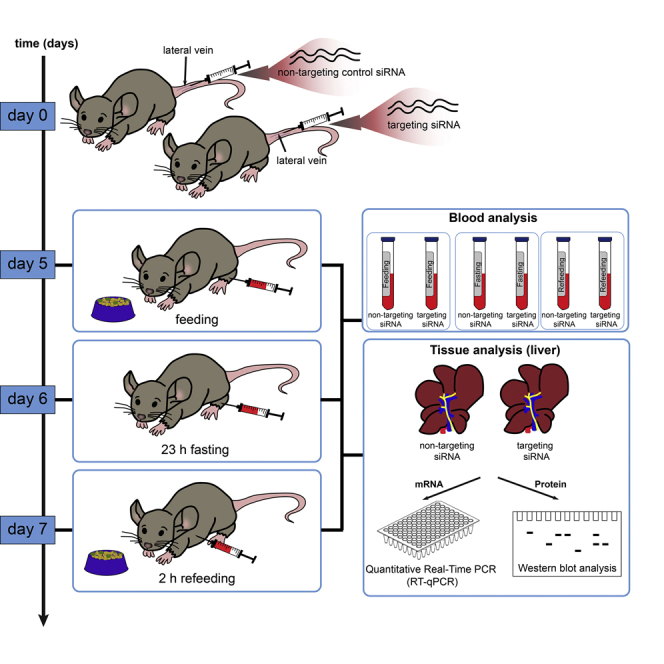

## Teaser

We present a protocol for in vivo siRNA-mediated knockdown of a gene of interest in mouse liver using systemic delivery via intravenous injection. We describe the step-by-step protocol for delivery of siRNA particles, with tips on how to optimize dosage. We detail steps for feeding/starving cycles as well as for liver tissue isolation, which is followed by gene expression analysis, measured at the mRNA and protein levels.

## Before you begin

### Selection of in vivo-specific oligonucleotides

The siRNA reagent can be obtained from a pre-designed collection provided by a company or could be custom designed. Usually, the user can choose from a few different pre-designed oligonucleotides targeting different regions of the transcript. In this protocol, the siRNA against *Usp9x* was chosen based on previous experiments performed in mouse embryonic fibroblasts, where the efficiency of siRNA-mediated knockdown of Usp9x could be assessed and compared for different oligonucleotides (data not shown). The amount of the negative control siRNA and the siRNA targeting the protein of interest needed for whole experiment should be calculated prior to ordering.

### Mice preparation

All mouse studies and procedures conformed to the ARRIVE guidelines (https://arriveguidelines.org/) under the jurisdiction of UK Home Office Project and Personal animal licenses and institutional guidelines.

***Note:*** Mouse colonies were bred and maintained in a specific pathogen-free “behind barrier” facility at the University of Cambridge. Mice were socially housed (3–5 mice per cage) on a 12 h light/dark cycle in individually ventilated cages with ad libitum access to food and water.

***Note:*** For initial testing, prepare small cohort of C57BL/6 J (Jackson Laboratories) male and female mice, aged between 7–10 weeks and weighing approximately 20–30*g*.

***Note:*** For the main study, prepare a cohort of at least 8–10 C57BL/6 J mice of each gender per condition.

### In vivo siRNA dose optimization in small cohort of mice

**Timing: 5**–**7 days**

The efficiency of the knockdown should be tested in a small group of mice prior to planning the proper experiment. In this protocol we aimed to knock down Usp9x, an essential deubiquitinating enzyme, in the liver tissue. Although intravenous siRNA injection does not allow for liver-specific siRNA delivery, most of the injected siRNA is taken up by this organ. Additionally, some siRNA uptake was observed in the spleen. In our protocol, we tested two different siRNA reagent doses of 1 mg/kg and 2 mg/kg body weight in mice. We analyzed liver tissue for knockdown efficiency on the protein level using a specific antibody at 5 and 7 days post-injection (injection day counted as day 0). For Usp9x, we obtained around 50% knockdown using 1 mg/kg and around 70%–80% using 2 mg/kg at 5 days post-injection (Figure 3A). We used 2 mg/kg across the whole final study. Usp9x is an essential gene, therefore we aimed for the shortest possible time of treatment to avoid any additional toxicity.

According to the data provided by the manufacturer (Ambion), the knockdown of the protein of interest can last up to 25 days post-injection. Readers should experimentally test the knockdown efficiency of their gene of interest independently, as knockdown efficiency depends on many factors, like protein half-life or mRNA expression level. Readers should also adjust the knockdown time to the purpose of their study and consider possible toxicity or side-effects caused by the knockdown of their gene of interest.

## Key resources table

**Table:** 

REAGENT or RESOURCE	SOURCE	IDENTIFIER
**Antibodies**		

Rabbit anti-USP9X (1:1000)	Abcam	Cat#ab19879; RRID:AB_470300
Mouse anti-Tubulin (1:3000)	Sigma-Aldrich	Cat#T9028; RRID:AB_261811
Mouse anti-GAPDH (1:3000)	Abcam	Cat#ab8245; RRID:AB_2107448

**Chemicals, peptides, and recombinant proteins**		

Invivofectamine 3.0 Reagent	Thermo Fisher	Cat#IVF3005
TRIzol reagent	Invitrogen	Cat#15596018
PureLink DNase Set	Invitrogen	Cat#12185010
RNase*Zap*™ RNase Decontamination Solution	Thermo Fisher	Cat#AM9782
96%–100% Ethanol	Sigma-Aldrich	Cat#V001229
Chloroform	Sigma-Aldrich	Cat#650498
cOmplete™ Protease Inhibitor Cocktail	Sigma-Aldrich	Cat#11697498001
Phosphatase Inhibitor Cocktail 2	Sigma-Aldrich	Cat#P5726
Phosphatase Inhibitor Cocktail 3	Sigma-Aldrich	Cat#P0044
PBS 1**×**	Invitrogen	Cat#10010-015
UltraPure RNAse/DNAse-free distilled water	Invitrogen	Cat#10977023

**Critical commercial assays**		

SuperScript III First Strand Synthesis System	Invitrogen	Cat#1880-051
PureLink RNA Mini Kit	Invitrogen	Cat#12183020
PowerUp™ SYBR™ Green Master Mix	Thermo Fisher	Cat#A25741
Pierce™ BCA Protein Assay Kit	Thermo Fisher	Cat#23225

**Experimental models: organisms/strains**		

Mouse: C57BL/6J	The Jackson Laboratory	Cat# JAX:000664; RRID:IMSR_JAX:000664

**Oligonucleotides**		

*In vivo* pre-designed siRNA targeting mouse USP9X: 5’- GGAUUACAGCUG GUAUUCAtt-3’	Ambion	Cat#S75828
Control siRNA	Ambion	Cat#4457289

**Software and algorithms**		

Excel	Microsoft Office	N/A
Prism v7	GraphPad	N/A

**Other**		

Fisherbrand™ Pre-Filled Bead Mill Tubes	Fisher Scientific	Cat#15555799
Precellys 24 tissue homogenizer	Bertin Instruments	Cat#P000669-PR240-A
1.7 ml Low Adhesion Hydrophobic Microcentrifuge Tubes	Bioquote	Cat#301726B190-10
RNase-free pipette tips	Starlab	Cat#variable
Microvette® CB300 K2E, Sarstedt	Sarstedt	Cat#16.444
Microvette® 100 K3E, Sarstedt	Sarstedt	Cat#20.1278
1 ml Syringes without needles	Terumo Science	Cat#SS+01T1
30G **×** ½ Inch needle	BD Microlance	Cat#304000
25G **×** 5/8 Inch (Agani needles)	Terumo Science	Cat#2516R1
23G **×** 1 Inch (Agani needles)	Terumo Science	Cat#2325R1

## Materials and equipment

### Preparation of siRNA stock solution

Before proceeding, convert the concentration of the siRNA stock to mg/mL (for example, use online calculator: https://horizondiscovery.com/en/ordering-and-calculation-tools/nmol-to-ug-calculator). In our study we prepared a 250 μM siRNA stock solution (Table 1).

• Briefly spin siRNA-containing tubes.
• Resuspend siRNA in UltraPure DNase/RNase-free distilled water and vortex briefly.
• Store the stock in small aliquots at −80^o^C.

**CRITICAL:** Use RNase-free tubes, RNase-free filter pipette tips and follow standard biological sterile techniques.

**Table 1:** Calculation of siRNA amount per injection (injection final volume is 200 μl) for two different doses (1 or 2 mg/kg body weight) using as an example: siRNA stock concentration 3.375 mg/mL=250 μM and mouse weight 20 *g*

example	mouse body weight	dose [mg/kg body weight]	siRNA amount [mg/mL] A=body wt **×** dose	amount of siRNA [volume in μl] B = $\frac{A}{3.375}\;$X 1000	siRNA conc. [μM] used for injection in a final volume 200 μl $\text{C}=\;\frac{250\;uM\;x\;B}{200\;ul}$
1	20 *g* = 0.02 kg	1 mg/kg	0.02 **×** 1= 0.02	5.92	7.4
2	20 *g* = 0.02 kg	2 mg/kg	0.02 **×** 2 = 0.04	11.85	14.8

### RIPA buffer preparation

**Table:** 

*RIPA buffer*		
Reagents	Final Concentration	Amount
Sodium chloride (5 M)	150 mM	1.5 mL
Triton X-100 (10%)	1%	5 mL
sodium deoxycholate (10%)	0.5%	2.5 mL
SDS (sodium dodecyl sulfate) (20%)	0.1%	250 μl
Tris, pH 8.0 (1M)	50 mM	2.5 mL
H_2_O	n/a	38.5 mL
**Total**	**n/a**	**50 mL**

**CRITICAL:** Sodium deoxycholate stock must be protected from light. RIPA buffer stock can be stored in aliquots at −20°C. Protease and phosphatase inhibitors must be added fresh to RIPA buffer just before use.

## Step-by-step method details

### Preparation of siRNA for injection

**Timing: 60**–**90 min (calculation time depends on the number of mice)**

In this protocol we used Invivofectamine™ 3.0 Reagent (ThermoFisher) and in vivo siRNA from Ambion. Alternatively, one could use in vivo-jetPEI® (Polyplus transfection) or AteloGene® (Bio-connect).

1. Calculating systemic amount and concentration of siRNA for a 1 mg/kg and 2 mg/kg dose.
  a. To dose each mouse according to its individual body weight (in mg/kg), the siRNA amount and its micromolar (μM) concentration has to be calculated.
  b. Before proceeding, convert the concentration of the siRNA stock to mg/mL (for example, use online calculator: https://horizondiscovery.com/en/ordering-and-calculation-tools/nmol-to-ug-calculator). In our study, we used 250 μM siRNA stock solution (Table 1).
  c. Weigh the mice a day prior to the experiment. This is necessary, as the Invivofectamine/siRNA complex takes 24 h to form.

***Note:*** In this protocol, we injected 200 μl per mouse. Always prepare **×**1.5 volume per injection as a backup in case of any spillage.

2. Preparation of Invivofectamine 3.0 - siRNA duplex complex mixture 24 h prior injection
  a. To determine the siRNA concentration for each individual mouse, we made use of the information worked out in Table 1 and kept all mouse records in an MS Excel file.
  b. The siRNA duplex solution for injection is prepared in accordance with the ThermoFisher protocol for Invivofectamine 3.0 Reagent Complexation with slight modifications.
  c. Mix siRNA duplex solution (as calculated in the column 5 of Table 1) with Complexation Buffer in 1:1 ratio.
  d. Thaw the Invivofectamine 3.0 reagent at 20°C–25°C and add to the diluted siRNA in 1: 0.5 ratio (modified from original manufacturer protocol).
  e. Vortex immediately to ensure Invivofectamine 3.0 complexation.
  f. Incubate invivofectamine 3.0- siRNA duplex mixture at 50^o^C, for 30 min.
  g. Spin the tubes briefly to collect the sample at the bottom of the tube.
  h. Dilute the sample by adding PBS, pH 7.4 (DNase RNase free) to a total volume of 200 μl.
  i. Store the solution for 24 h at 4°C.

**CRITICAL:** Use RNase-free tubes, RNase-free filter pipette tips and follow standard biological sterile techniques.

### Mouse intravenous (IV) administration

**Timing: 10 min for heating up, 5**–**15 min for injection per mouse.**

Intravenous (IV) administration in mice is usually performed via the lateral (right and left) tail veins.

**CRITICAL:** Tail vein injection is technically difficult, particularly in mice on a C57BL/6 J background, due to their relatively scaly skin and darker tail. Adequate training prior to the experiment, preferably in albino background mice, could be useful.

3. Randomize mice prior to the treatment and match them by weight and gender.
4. Place the mice in a heating chamber (Figure 1A) at 38^o^C for 8–10 min to dilate the tail vein.

**Figure 1:**
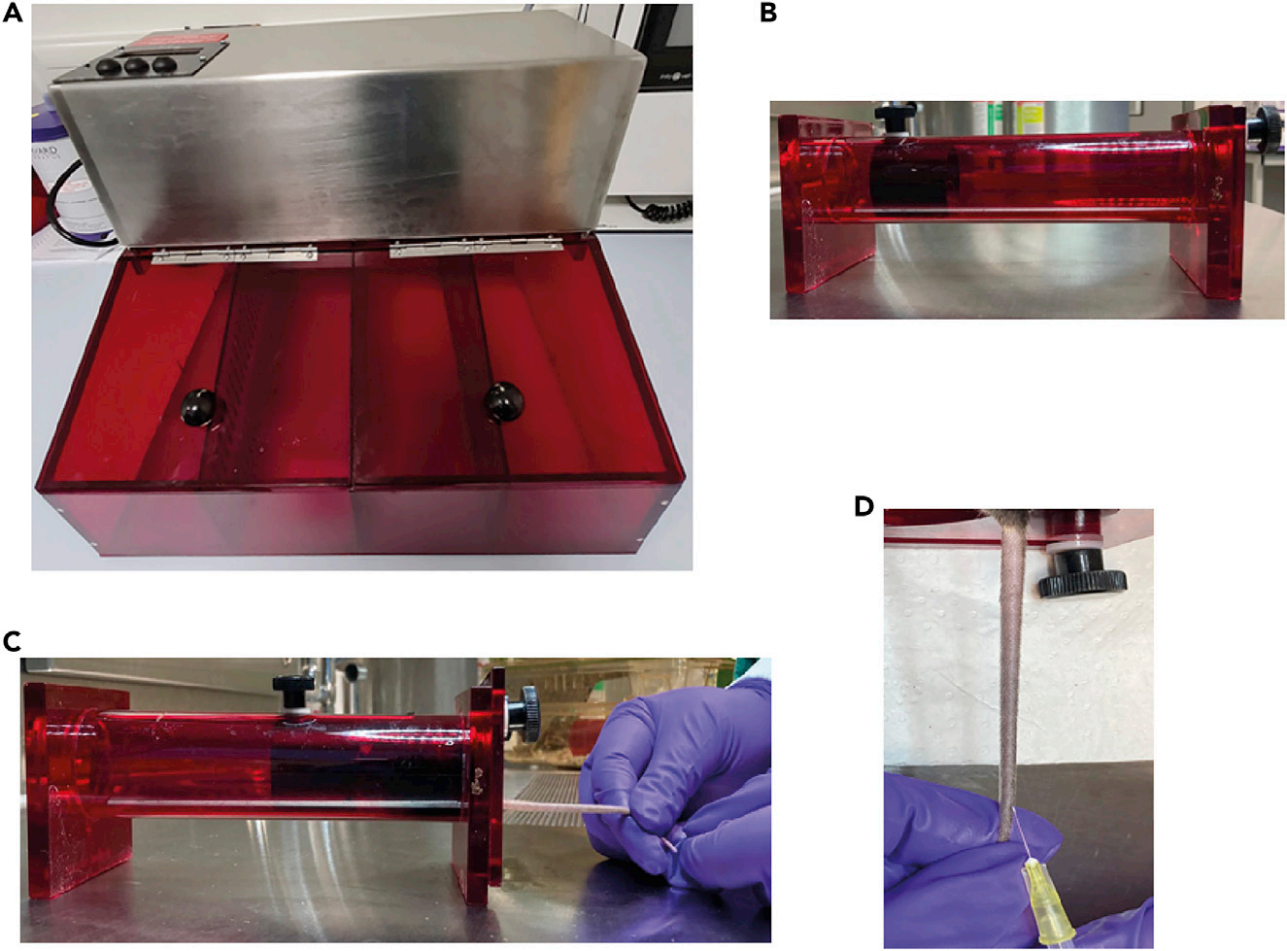
Mouse Intravenous (IV) administration (A) Heating Box (Chamber). (B) Restrainer without mouse. (C) Restrainer with a mouse. (D) Tail vein injection
5. Restrain mice using the mechanical restraint device with the pores in the front to ensure that the mouse is breathing and use the holes in the back to pull out the mouse tail (Figures 1B and 1C).

**CRITICAL:** The restraining device should be of appropriate size for the mice to be injected. After use, the restraining device should be cleaned thoroughly to avoid pheromone-induced stress or cross-infection.

6. Disinfect the tail with antiseptic solution (75 % ethanol).
7. Rotate or turn the restrainer to access the lateral (either left or right) tail vein and immobilize the tail with the left hand.
8. Visualize the vein. If the vein is not visible, leave the mouse in the heated box for another 5 min. Troubleshooting 1
9. Tightly press the tail and orient the needle (with 1 mL injection syringe ) in your right hand parallel to the tail, with the beveled edge of the needle facing up.
10. Insert needle (30G) into the vein starting at the tip of the tail (distally) at about a 30° angle (Figure 1D).

**CRITICAL:** Always start injection by placing the needle at the distal end of the tail vein. In case of an unsuccessful injection, a second attempt can be made at a relatively more proximal site. Alternatively, the second vein on the other side (could be left or otherwise) can be attempted.

11. If the needle advances in the blood vessel, there will not be any resistance and the compound will flow easily during administration. In a case of unsuccessful needle injection, the material from the syringe will not move through the vein or it will cause blanching around the vein.
12. Administer compound slowly and evenly.
13. Upon needle retrieval, immediately apply gentle pressure with gauze (30–60 seconds) on the vein until bleeding has stopped. Animals should not be returned to their cage before the blood flow has stopped.
14. Monitor animal for 5 to 10 min to ensure hemostasis.
15. In our protocol, mice were left for 5–7 days post injection prior to tissue analysis.

**CRITICAL:** After the siRNA injection, animals should be monitored daily to observe any side effects caused by either siRNA injection (should be equal for both control and gene of interest siRNAs) or by loss of your specific gene of interest (should not be observed in control animals).

### Blood sampling

For blood sampling from superficial vein **Timing: 10**–**15 min per mouse.**

For terminal blood collection **Timing: Duration of cardiac puncture is 5**–**10 min per mouse.**

We used this method to collect blood samples before and after a fasting period to monitor the levels of different metabolites as well as markers of liver damage in the blood plasma.

16. Blood sampling from superficial vein
  Prior to fasting period, a blood sample was collected from a superficial vein. Sampling from the lateral saphenous vein is a relatively quick and easy method for obtaining a small amount of blood from a mouse. In this method, conscious mice are restrained using a restraint tube and approximately 100–120 μl of blood is collected.
  a. Restrain a conscious mouse using a restraint tube (optionally, one can use a home-made 50 mL Falcon tube restrainer with a hole cut at the bottom to allow the mouse to breath).
    **CRITICAL:** Restraint tube should be appropriate for the size of the mouse. All forms of restraining equipment should be frequently washed to prevent pheromone-induced stress or cross-infection.
  b. To collect blood, the hind leg should be stretched out using your left-hand index finger and thumb, while the other fingers are used as barrier to stop the mouse escaping from the restrainer tube. Immobilize the leg in an extended position by applying gentle downward pressure with fingers immediately above the knee joint.
  c. Shave the fur on the stretched back of the leg (or thigh) using an electric hair clipper. The vein should be visible on the skin after shaving.
  d. Disinfect the vein with antiseptic solution (75 % ethanol).
  e. Collect the blood using a 25G needle into anticoagulant-treated EDTA tubes (Microvette® CB300 K2E, Sarstedt).
  f. Stop the blood flow by gentle finger pressure with sterile tissue paper or gauze over the puncture site. Animals should not be returned to their cage before the blood flow has stopped.
  g. Centrifuge blood samples for 10 min at 1,000–2,000 **×** *g* at 4°C and collect the supernatant plasma to a new tube and store on dry ice (long term storage in −20°C or lower).
  h. In this protocol, blood plasma was analyzed for basal levels of glucose, insulin, and for liver function tests by the Core Biochemical Assay Laboratory (CBAL), a Cambridge University Hospital facility.

**CRITICAL:** The number of attempts for blood sampling should be minimised (no more than three needle sticks in any one attempt). There is always a possibility that a mouse may not bleed after three attempts to access the saphenous vein. Try to relax the mouse leg by tapping its foot gently, as this may allow a small amount of blood (10–20 μl) to be collected immediately.

17. Terminal blood collection
  After the fasting period, a terminal blood sample was collected using cardiac puncture, a suitable technique that allows one to obtain a single, large, good quality blood sample from a mouse under deep terminal anesthesia. Depending on the size of the mouse and whether the heart is kept beating, 0.1 - 1 mL of blood can be obtained from each mouse.
  a. Anaesthetize mouse using isoflurane in an isoflurane chamber/box.
  b. Once the mouse is in deep anesthesia (checked by pinching its toe with your fingernails), place it in a lying position with the chest and abdomen side up (ventral side up) on the table / or on the operating bench with the isoflurane mask on.
  c. Prepare a 1 mL syringe with a 23G needle (with plunger in).
  d. The heart is located approximately at the level of the elbow on the left. The point of the needle entry or heart puncture is measured against the point of the elbow on the chest wall.
  e. Insert the needle (bevel-up) at an angle of slightly more than 45^o^ at a point midway on the chest wall dorsoventrally, to enable the heart to be punctured.
  f. Apply slight back pressure with the syringe. If the needle is in the heart, blood will flow into the syringe.
  g. Wait until blood has filled the syringe before adding additional back pressure on the syringe. Avoid applying back pressure and releasing it repeatedly, as this can cause clotting in the syringe.
    **CRITICAL:** Blood should be withdrawn slowly to prevent the heart collapsing.
  h. Collect blood by capillary action into anticoagulant-treated EDTA tubes (Microvette® CB300 K2E, Sarstedt).
  i. After blood collection, the mouse is culled by cervical dislocation.
  j. Centrifuge blood samples for 10 min at 1,000–2,000 **×** *g* at 4°C and transfer supernatant plasma to a new tube and store on dry ice (long term storage in −20°C or lower).
  k. In this protocol, blood plasma was analyzed for basal levels of glucose, insulin, and for liver function by the Core Biochemical Assay Laboratory (CBAL), a Cambridge University Hospital facility.

### Mouse food deprivation

**Timing: Duration of food deprivation is 23 h, with 1**–**2 h for weighing the mice, preparing the cages, and keeping records**.

The body weight of each mouse should be measured before and after the fasting cycle and recorded.

In our laboratory, we routinely use two fasting periods: 23 h and 46 h. The 46 h period consists of a 22 h fasting period followed by refeeding for 2 h in order to synchronize mice in each group (since mice feed freely under normal conditions), and then a subsequent period of fasting for 22 h. Food deprivation typically starts at midday (from 12:00 to 1:00pm) and lasts untilthe next morning (by 10:00–11:00 am).

18. For food deprivation, mice are transferred to a fresh cage without food, either singly or doubly housed. For fasting, instead of removing food from the old cage, move the mice to a fresh cage without food to avoid any food intake causing experimental error, e.g., in case any food particles were on the floor or nesting of the old cage.
19. During the food deprivation period, mice have free access to water throughout the procedure.
20. In the experiments where the response to insulin signaling was assessed, the 23 h fasting period was followed by a refeeding period for 2 h to cause a synchronized-rise in glucose and insulin levels in the blood.
21. After 2 h of refeeding, mice were weighed to ensure they have eaten the food.
22. After the desired fasting/refeeding cycle, mice were anaesthetized using isofluorane and terminal blood samples were collected by cardiac puncture. After death has been confirmed by cervical dislocation, the liver and other tissues were collected.

**CRITICAL:** Mice will lose weight in this protocol. If the weight loss exceeds 15%, the mouse should be immediately either culled or re-fed (according to our licence).

### Tissue (liver) dissection

**Timing: 5**–**10 min per mouse.**

**Figure 2:**
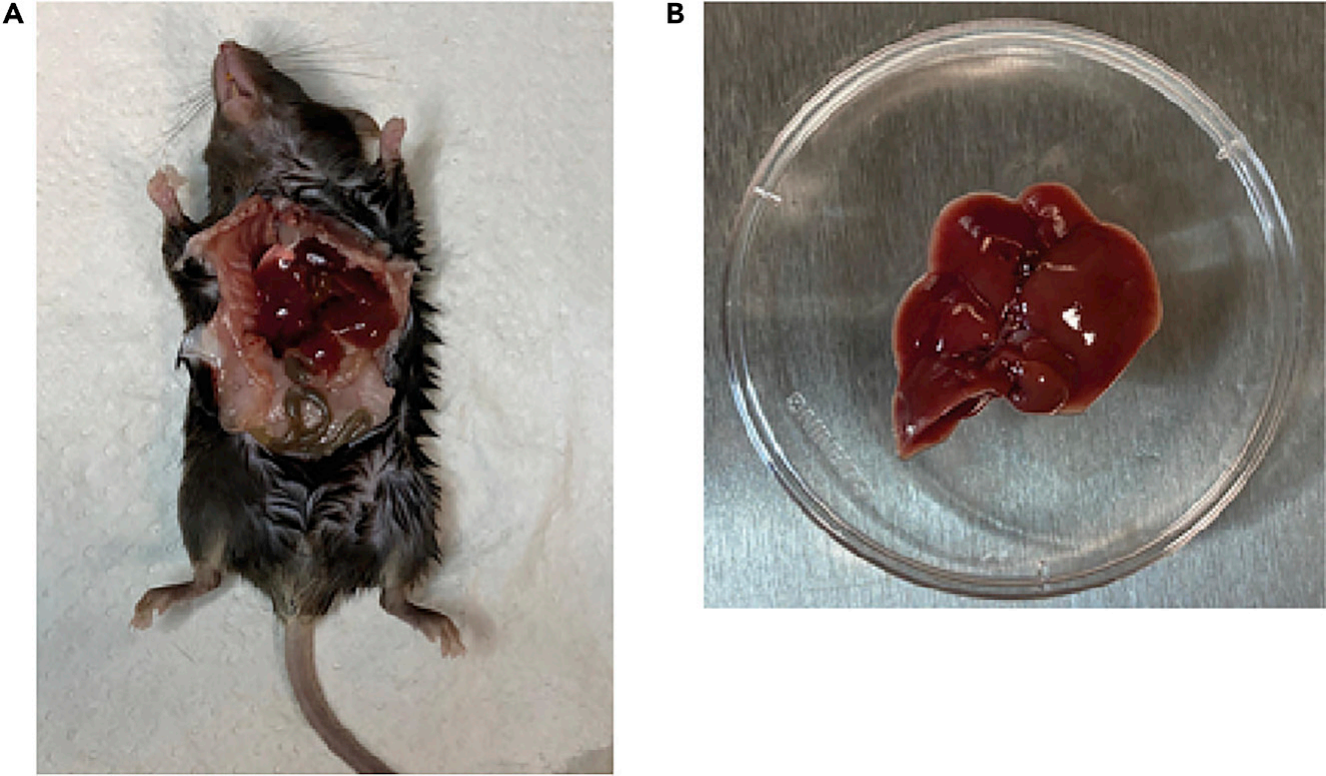
Liver dissection (A) Mouse open abdomen. (B) dissected liver.
25. Rinse liver in cold 1**×**PBS and immediately store the liver lobes on dry ice.
26. Transfer mice tissue (in this case liver) into the −80°C freezer for long term storage.

***Note:*** In the trial experiment performed with a small mice cohort, additional organs such as brain, pancreas, spleen, muscle, and kidney should be collected for analysis. This will help the investigator assess if the protein of interest was also targeted in organs besides the liver.

23. Sanitize the mouse abdomen surface with 70 % ethanol.
24. Use scissors and tweezers to open the abdomen and dissect out the entire liver (Figure 2).

### RNA isolation and analysis of mRNA levels

**Timing: 4**–**6 h**

27. Prepare homogenate from frozen liver tissue.
  a. Prepare a box with dry ice and a box with ice, and a scalpel and forceps cleaned with 70% ethanol and RNase decontamination solution.
  b. Using scalpel and forceps, cut approximately 50–100 mg of the liver tissue.
    ***Note:*** It is important to cut the same liver lobe from each tissue sample. For protein isolation, cut a piece from the same tissue area. Do not allow thawing of the entire tissue. Freezing is not obligatory and one can analyse fresh tissue – the freezing step enables analyses at convenient times after the dissection.
  c. Place a liver fragment into a pre-cooled (dry ice) 2 mL RNase- and DNase-free homogenizing tube containing ceramic beads. Keep the tube on dry ice to prevent thawing, while preparing following samples. In case liver tissue fragments were prepared fresh, continue to step d.
  d. Once all tissues are cut, place tubes on ice, add 1 mL of TRIzol reagent per sample and homogenize using a homogenizer (work in the cold room).
    ***Note:*** TRIzol is highly toxic - always work in a fume hood when working with TRIzol.
  e. Incubate the lysate for 5 min at 20°C–25°C (room temperature) and centrifuge at 12,000 **×** *g* for 10 min at 20°C.
  f. Transfer supernatant to new 1.5 mL RNase-free tubes.

**Pause point:** Homogenized samples can be stored at –60 to –80°C for at least one month with no noticeable differences in RNA yield, compared with fresh samples.

28. Purify RNA using PureLink®RNA kit and on-column PureLink® DNase treatment, following manufacturer’s protocol specific for TRIzol extraction (starting on page 49 in manufacturer’s manual). Purified RNA can be stored long-term at −80°C.
29. Synthetize cDNA using SuperScript III First Strand Synthesis System, following the standard manufacturer protocol. cDNA can be stored long-term at −20°C.
30. Perform real-time PCR analysis with prepared cDNA using PowerUp™ SYBR™ Green Master Mix, following standard manufacturer protocol. Troubleshooting 3

***Note:*** In the real-time PCR assay, CT values are specific to your gene of interest. The ΔCT value shows a difference in expression between your gene of interest and a reference gene (usually housekeeping gene; in our protocol we used mouse *Gapdh*). The ΔΔCT shows a fold change difference between the control and treated sample. When comparing two (or more) groups of mice, for example control siRNA vs. siRNA knockdown or fed vs. starved mice, always prepare one sample with an equal mixture of each cDNA from all control/basal conditions mice in the experiment. The ΔCT result for this sample will serve as a normalization factor allowing one to calculate fold-change in the expression of your gene of interest (ΔΔCT) for each mouse (both control and treated).

### Protein isolation and analysis of protein levels

**Timing: 2**–**4 h**

31. Prepare homogenate from a frozen liver tissue.
  a. Prepare a box with ice, and scalpel and forceps cleaned with ethanol.
  b. Using scalpel and forceps, cut approximately 100 mg of the liver tissue.
    ***Note:*** It is important to cut the same liver lobe from each tissue sample. For protein isolation, cut a piece from the same tissue area. Do not allow thawing of the entire tissue. Freezing is not obligatory and one can analyse fresh tissue – the freezing step enables analyses at convenient times after the dissection.
  c. Place a liver fragment into a pre-cooled 2 mL tube with 1 mL ice-cold 1**×**PBS and wash the liver fragment by inverting the tube a few times.
  d. Transfer the liver fragment to a pre-cooled 2 mL tube containing ceramic beads and 500 μl of cold RIPA buffer (kept on ice) with protease and phosphatase inhibitors. Keep the tube on ice, while preparing following samples.
    ***Note:*** RIPA buffer can be prepared earlier and stored in aliquots in −20°C; always add protease and phosphatase inhibitors freshly prior to use.
  e. Once all tissues are prepared, homogenize using a homogenizer.

32. Incubate homogenates on ice for 30 min and centrifuge 12,000 **×** *g* for 10 min at 4°C.
33. Transfer supernatants into fresh pre-cooled eppendorf tubes and keep on ice.
34. Determine protein concentration using Pierce™ BCA Protein Assay Kit following manufacturer’s protocol.

**Pause point:** Homogenized samples can be stored long-term at –80°C. Prepare aliquots to avoid multiple freeze-thaw cycles.

35. Analyze the protein levels and post-translational modifications (for example protein phosphorylation) using SDS-PAGE and Western blotting using specific antibodies. Troubleshooting 5
36. Normalize your protein-of-interest levels (in our case Usp9x) to the levels of a house-keeping gene, like mouse Tubulin or Gapdh. Present the data as ratios of protein-of-interest signal intensity to signal intensity of a house-keeping gene using arbitrary units (AU). Test a few house-keeping genes and stain the protein ladder resolved by SDS-PAGE gel using Coomassie staining, to ensure equal protein loading across samples. Troubleshooting 2, Troubleshooting 4

***Note:*** Often, the number of samples that one is analysing exceeds the number of wells available on one gel. To be able to compare the protein levels across all animals, load the same control sample (or few common controls) onto each gel. The use of common controls on different gels allows normalization, so one can compare large numbers of samples.

## Expected outcomes

### Protein knockdown efficiency in the liver tissue – western blot analysis

**Figure 3:**
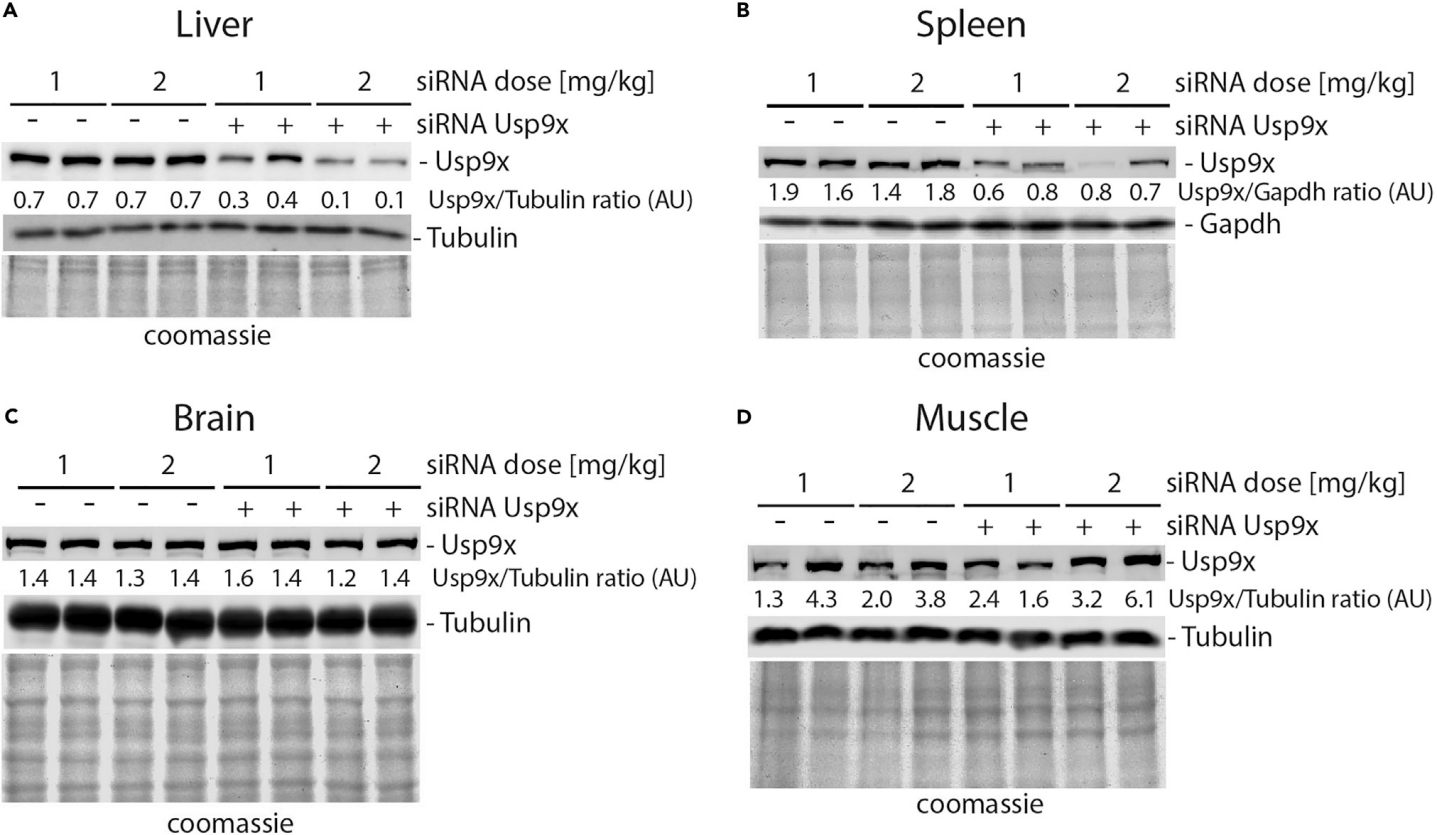
Protein knockdown efficiency in the liver tissue – western blot analysis Mice were injected with 1 mg/kg or 2 mg/kg dose of non-targeting or siRNA against *Usp9x*. Two mice per condition were used. On day 5 post siRNA injection, liver (A), spleen (B), brain (C) and muscle (D) were dissected. Tissue lysates were analyzed by SDS-PAGE and Western blotting. Protein levels were determined by immunoblotting using specific antibodies for Usp9x, Tubulin or Gapdh. Total protein load on gel was stained using Coomassie blue; arbitrary units (AU).

In the dose optimization trial experiment, we obtained around 50% knockdown using 1 mg/kg dose and around 70%–80% using 2 mg/kg dose at 5 days post-siRNA injection (Figure 3A). We also observed up to 30% knockdown in spleen, most probably connected to its function as a blood filtering organ (Figure 3B). We did not observe any decrease of Usp9x in the brain or muscle tissue lysates (Figures 3C and 3D). We used 2 mg/kg dose across the whole final study.

### ALT test to monitor possible liver damage caused by knockdown

The ALT test measures the levels of alanine aminotransferase (ALT) in the blood. High levels of ALT in the blood indicate liver damage (for example experiment, please refer to the original publication (Wrobel et al., 2020).

### Glucose and insulin levels

Measuring the levels of glucose and insulin in mouse blood samples after 23 h fasting and 2 h refeeding period allows one to confirm food uptake and assess correct stimulation of insulin release by the pancreas in the control and mice treated with targeting siRNA (for example experiment please refer to the original publication (Wrobel et al., 2020)).

## Limitations

Systemic delivery of siRNA by intravenous injection allows for an effective knockdown mostly in the liver tissue. siRNA injected intravenously was shown to be targeted not only to the liver, but also to kidney, spleen or pancreas (Larson et al., 2007; Thompson et al., 2012). This might cause more generalized phenotypes arising from targeting of the protein of interest in different organs. Therefore, caution must be taken when interpreting data.

## Troubleshooting

### Problem 1

Mouse is not fully dosed with siRNA solution, due to technical errors while doing IV injections

### Potential solution

The mouse line used in this study is based on C57BL/6 J strain, which makes IV injection difficult, as the veins contrast only faintly in relation to the skin. Vein visibility can be improved by utilizing a dedicated illumination device. Additionally, the IV procedure may be facilitated by optimizing a mouse restrainer or by further extending pre-warming of the animal to improve vein visualization.

Keep a record of each mouse dose and always refer to it while analyzing the results.

### Problem 2

Knockdown was not observed

### Potential solution

If no difference in the levels of protein of interest is observed during the optimization step with either of siRNA doses, the knockdown time can be extended for up to 20–25 days. If knockdown is still not observed, additional targeting siRNAs should be tested.

### Problem 3

Efficient knockdown is observed at the level of a protein, but not at the mRNA level

### Potential solution

If the knockdown is only observed at the levels of a protein, but not its mRNA, it still confirms that the procedure was successful. In this study, we confirmed specific knockdown of Usp9x protein, but we did not observe any significant decrease in its mRNA levels in liver tissue from mice treated with siRNA against *Usp9x*. This unexpected observation may be explained by translational repression, where the siRNA downregulates translation of the target mRNA without inducing detectable mRNA cleavage and reducing mRNA levels (Wrobel et al., 2020; Wu and Belasco, 2008; Zeng et al., 2003).

### Problem 4

Knockdown efficiency is low

### Potential solution

In this protocol, we analyzed liver issues isolated from mice treated with non-targeting siRNA or siRNA against *Usp9x* at days 5, 6 and 7 post-injection. For each time point, we observed a knockdown efficiency of around 60%–80% across a group (Figure 3A and Wrobel et al. 2020). Usp9x is an essential gene, therefore we aimed for the shortest possible time of treatment to avoid any additional toxicity. According to the data provided by the manufacturer (Ambion), the knockdown of the protein of interest can last up to 25 days post injection. Readers should experimentally test the knockdown efficiency of their gene of interest independently, as knockdown efficiency depends on many factors, like protein half-life or mRNA expression level. Readers should also adjust the knockdown time to the purpose of their study and consider possible toxicity/side-effects caused by the knockdown of their gene of interest.

### Problem 5

High variation in the levels of targeted protein in both control and siRNA-targeted liver samples

### Potential solution

During liver tissue dissection and lysate preparation, it is critical to use the same liver lobe and region across all samples. The liver has several lobes, each with reported physiological variations, which may contribute to molecular differences– for example, in the expression pattern of genes reflected in the differences in the protein level pattern. To minimize the variation in the levels of protein or mRNA between mice, always identify and cut the same region of a tissue across all mice in the experiment.

## Resource availability

### Lead contact

Further information and requests for resources and reagents should be directed to and will be fulfilled by the lead contact, David C Rubinsztein (dcr1000@cam.ac.uk).

### Materials availability

All unique/stable reagents generated in this study are available from the lead contact with a completed Materials Transfer Agreement.

### Data and code availability

This study did not generate datasets or code.
